# Comparison Between a New PSA Assay With the Well‐Established Beckman Coulter Immunoassay: A Preliminary Report

**DOI:** 10.1002/ansa.70017

**Published:** 2025-05-14

**Authors:** Evelina La Civita, Mariano Fiorenza, Giuseppe Jannuzzi, Carmela Polito, Rosa Sirica, Gianluigi Carbone, Domenica Sorvillo, Aniello Saviano, Matteo Ferro, Daniela Terracciano

**Affiliations:** ^1^ Department of Translational Medical Sciences University of Naples “Federico II” Naples Italy; ^2^ Unit of Urology Department of Health Science University of Milan ASST Santi Paolo and Carlo Milan Italy

**Keywords:** immunoassays, prostate cancer, PSA

## Abstract

Prostate‐specific antigen (PSA) is a critical biomarker for prostate cancer (PCa) patient clinical management. In this study, we sought to compare three different immunoassays (CLIA): Beckman Coulter Access Hybritech (PSA‐B) (reference method), Immulite 2000 PSA (PSA‐I) and the newly introduced Atellica IM PSA assay (PSA‐A). We selected serum samples from our routine clinical testing at University Hospital Federico II between April and May 2024 from 104 men with a median age of 66 years (interquartile range = 57–74). Total PSA was determined using three different assays: PSA‐B, PSA‐A and PSA‐I. A significant correlation between PSA‐B and PSA‐I assays was found for samples in the overall population (Spearman *r* (*ρ*) = 0.99, *p* < 0.0001). PSA‐I displayed a strong correlation with PSA‐B for values below 2 ng/mL (*ρ* = 0.98, *p* < 0.0001), for values between 2 and 10 ng/mL (*ρ* = 0.97, *p* < 0.0001) and for values above 10 ng/mL (*ρ* = 0.77, *p* < 0.0001). A significant positive correlation was found between PSA‐B and PSA‐A in the overall population (*ρ* = 0.97, *p* < 0.0001) and in stratified analyses between PSA‐A and PSA‐B for values below 2 ng/mL (*ρ* = 0.86, *p* < 0.0001), from 2 ng/mL to 10 ng/mL (*ρ* = 0.93, *p* < 0.0001) and above 10 ng/mL (*ρ* = 0.77, *p* < 0.0001). Although both PSA‐I and PSA‐A demonstrated a significant positive correlation with PSA‐B, PSA‐I displayed a significantly better correlation with PSA‐B than PSA‐A in samples with PSA below 2 ng/mL.

## 1 Introduction

Circulating prostate‐specific antigen (PSA) has been widely used for early detection and follow‐up of prostate cancer (PCa) patients over last decades [1].

Following the purification of PSA in 1979 [2], several immunometric assays were developed to measure PSA in serum, targeting both PSA complexed with α1‐antichymotrypsin and free PSA [3].

The free‐to‐total PSA ratio has been widely used to distinguish benign from malignant prostatic diseases; however, due to its suboptimal diagnostic performance in the identification of clinically significant PCa [4], other laboratory tests, such as Phi, 4Kscore and Stockholm3, have been developed [5]. Nevertheless, PSA remains a pillar in PCa patient clinical management [6], despite notable drawbacks, such as the lack of assay interchangeability due to the non‐uniformity of various manufacturers' assays [7, 8]. The variability of results from different assays could affect interpretation of PSA values [9].

PSA value inconsistency is mainly due to different assay architecture and measurement technologies with dissimilar antibody epitope specificities and affinities, leading to non‐uniform assay calibration and non‐equimolar measurement of PSA [10, 11]. Although the bias between different PSA assays has notably decreased after the World Health Organization (WHO) introduced reference standards for total and free PSA in 1999 [10], several authors showed that PSA results are still not interchangeable across manufacturers [12, 13]. Against this background, we compared the newly introduced Atellica (Siemens) PSA assay with the commonly used PSA Immulite assay (Siemens) and with the gold standard PSA Beckman assay to provide a timely and updated overview of potential implications of PSA discrepancies in the biopsy decision‐making based on PSA values.

## 2 Materials and Methods

### 2.1 Serum Samples

We selected serum samples from our routine laboratory activity at University Federico II Hospital between April and May 2024 from 104 men with a median age of 66 years (interquartile range = 57–74). Samples showing evidence of haemolysis, icterus or lipemia were excluded. Three aliquots (500 µL) were preparedfor each sample within 2 hours of blood collection and stored at −80°C to ensure stability until analysis.

### 2.2 Assays

Total PSA was determined using three different immunoassays (CLIA), including Access Hybritech (PSA‐B) (Beckman Coulter), Immulite 2000 PSA (PSA‐I) (Siemens) and Atellica IM PSA assay (PSA‐A) (Siemens). All measurements were performed in one single run, using a single lot of reagents for each platform. The characteristics of analytical methods are shown in Table 1. Determinations were made on the Beckman Coulter Access 2 immunoassay, the Immulite 2000 and the Atellica IM analysers. Testing was performed according to the manufacturer's instructions. Quality control materials from the Bio‐Rad system (Bio‐Rad Laboratories Inc., Irvine, CA, USA) were used. Analytical measurement ranges (AMR) of serum PSA ranged from 0.008 to 150 ng/mL for PSA‐B, from 0.01 to 100.00 ng/mL for PSA‐A and from 0.04 to 150.00 ng/mL for PSA‐I.

**TABLE 1:** Analytical methods characteristics.

Analyser	Beckman Coulter	Atellica IM	Immulite 2000
Kit	Access Hybritech PSA	Atellica IM PSA	Immulite 2000 PSA
Measuring range	0.008–150 ng/mL	0.01–100 ng/mL	0.04–150 ng/mL
Absence of Hook effect until (as noticed in the insert kit)	50000 ng/mL	8000 ng/mL	4277 ng/mL
Test principle	Sandwich immunoassay in one step	Sandwich immunoassay in one step	Sandwich immunoassay in one step

### 2.3 Method Comparison and Statistical Analysis

We performed pairwise comparisons between PSA‐B, PSA‐I, and PSA‐A using 104 serum samples. The range of the selected serum samples, as measured by the Immulite method routinely used in clinical practice, ranged from 0.036 to 143.0 ng/mL. PSA‐B was used as the reference method for statistical analysis. For comparison of different methods, we calculated the Spearman coefficient ρ (rho) we calculated the Spearman correlation coefficient (ρ) for each assay pair. To assess the statistical significance of the differences in each correlation, we used the Fisher test. In addition, we used the Bland–Altman method to evaluate the agreement between the measurements. The study design and method comparison were aligned with CLSI EP09‐A3 guidelines.

## 3 Results

### 3.1 Comparison of PSA‐B and PSA‐I

**FIGURE 1:**
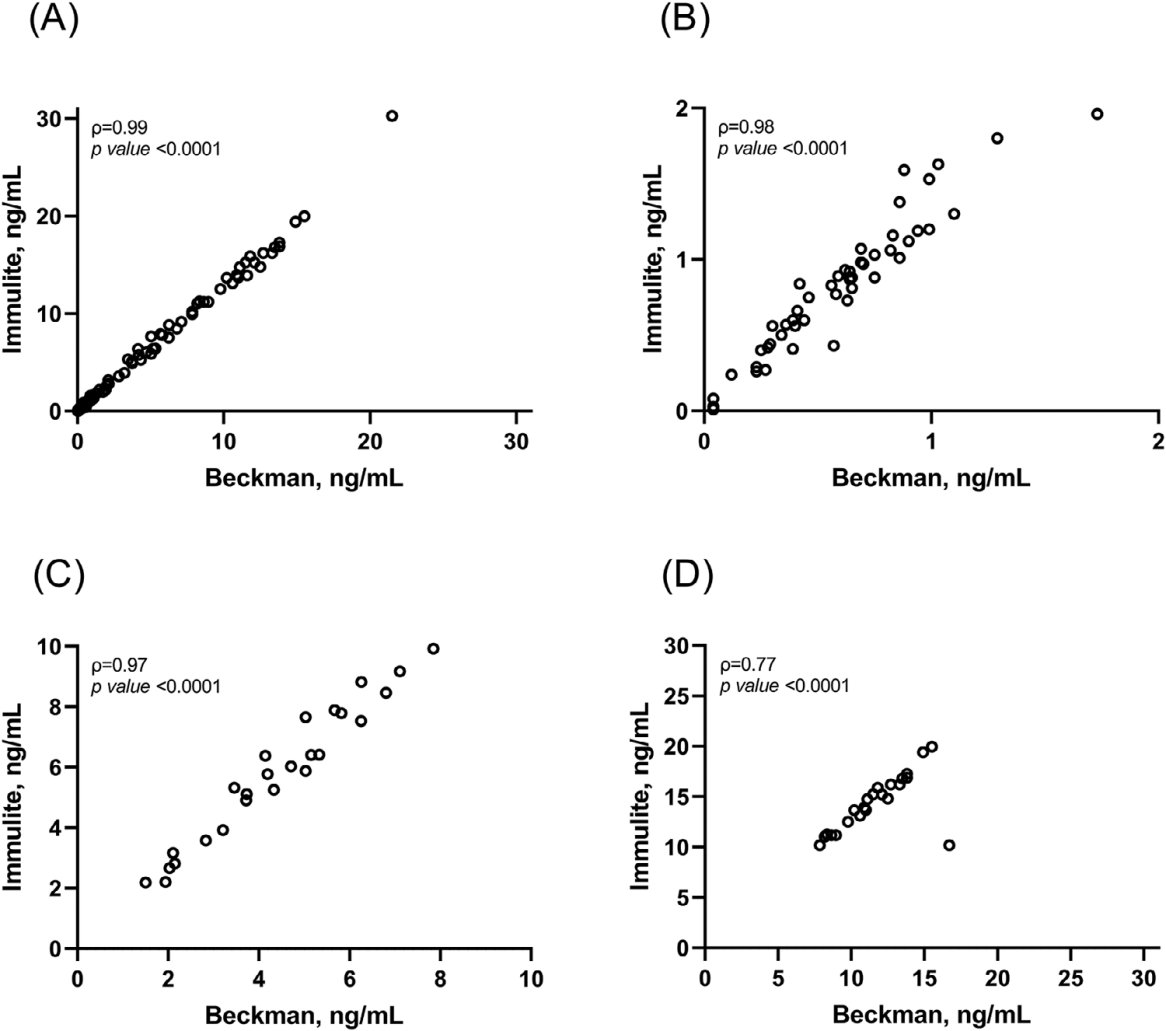
Spearman's correlation analyses between PSA‐B and PSA‐I in the overall population (A), for samples below 2 ng/mL (B), for values ranging from 2 to 10 ng/mL and for values above 10 ng/mL.

As shown in Figure 1A, a very strong correlation between PSA‐B and PSA‐I assays was observed in the overall population (*ρ* = 0.99, *p* < 0.0001). To further understand the agreement of PSA‐I with the reference method, we analysed the correlation between PSA‐B and PSA‐I by stratifying values into clinically relevant ranges. As shown in Figure 1B,C, PSA‐I showed excellent correlation with the reference method for values below 2 ng/mL (*ρ* = 0.98, *p* < 0.0001) and for values between 2 and 10 ng/mL (*ρ* = 0.97, *p* < 0.0001). A strong correlation between PSA‐B and PSA‐I was found for values above 10 ng/mL (*ρ* = 0.77, *p* < 0.0001) (Figure 1D).

### 3.2 Comparison of PSA‐B and PSA‐A

**FIGURE 2:**
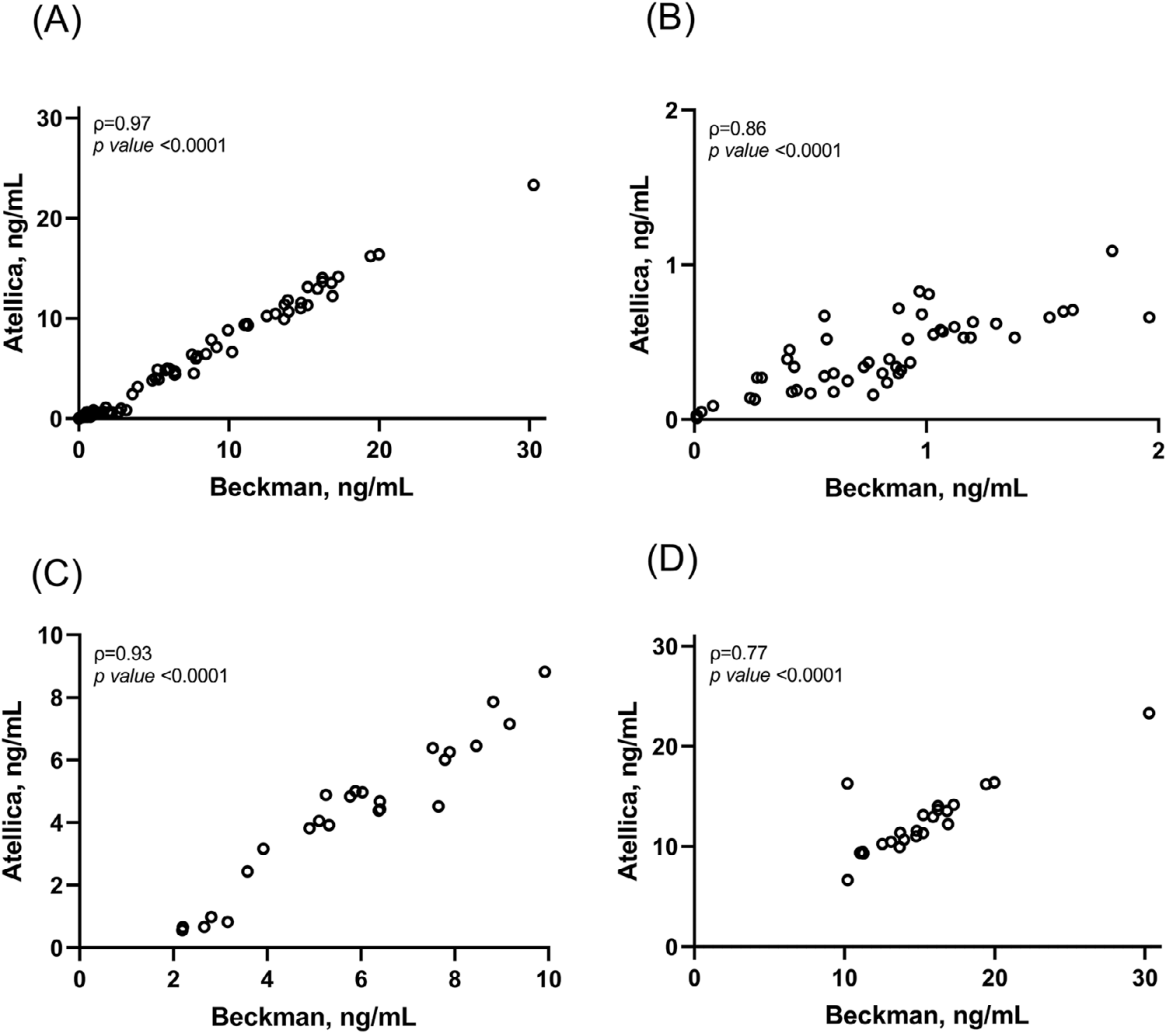
Spearman's correlation analyses between PSA‐B and PSA‐A in the overall population (A), for samples below 2 ng/mL (B), for values ranging from 2 to 10 ng/mL and for values above 10 ng/mL.

As shown in Figure 2A, a strong and statistically significant correlation between PSA‐B and PSA‐A was found for samples in the overall population (*ρ* = 0.97, *p* < 0.0001). In addition, strong correlations were found for samples below 2 ng/mL (*ρ* = 0.86, *p* < 0.0001) (Figure 2B) and for samples ranging from 2 to 10 ng/mL (*ρ* = 0.93, *p* < 0.0001) (Figure 2C), whereas a moderate correlation between PSA‐B and PSA‐A was found for samples above 10 ng/mL (*ρ* = 0.77, *p* < 0.0001) (Figure 2D). Although PSA‐A showed strong agreement with the reference method, the correlation between PSA‐I and PSA‐B was significantly higher than the correlation between PSA‐A and PSA‐B for samples below 2 ng/mL (*ρ* = 0.98 vs. *ρ* = 0.86; *p* < 0.001).

### 3.3 Comparison of PSA‐I and PSA‐A

**FIGURE 3:**
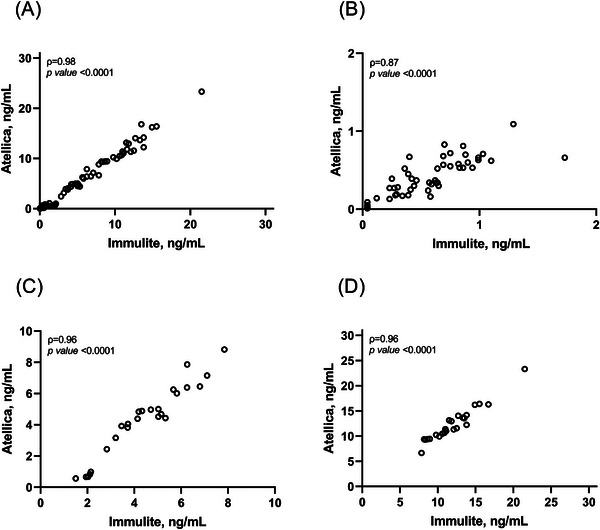
Spearman's correlation analyses between PSA‐I and PSA‐A in the overall population (A), for samples below 2 ng/mL (B), for values ranging from 2 to 10 ng/mL and for values above 10 ng/mL.

As shown in Figure 3A PSA‐I and PSA‐A displayed a significant positive correlation for the overall population (*ρ* = 0.98, *p* < 0.0001) as well as for samples below 2 ng/mL (*ρ* = 0.87, *p* < 0.0001), for samples ranging from 2 to 10 ng/mL (*ρ* = 0.96, *p* < 0.0001) and for samples above 10 ng/mL (*ρ* = 0.96, *p* < 0.0001).

### 3.4 Concordance Between PSA‐B With PSA‐I and PSA‐A

The overall concordance with PSA‐B in identifying values below 0.2 ng/mL was 100% for both PSA‐I and PSA‐A.

### 3.5 Comparability of PSA‐I and PSA‐A With PSA‐B

**FIGURE 4:**
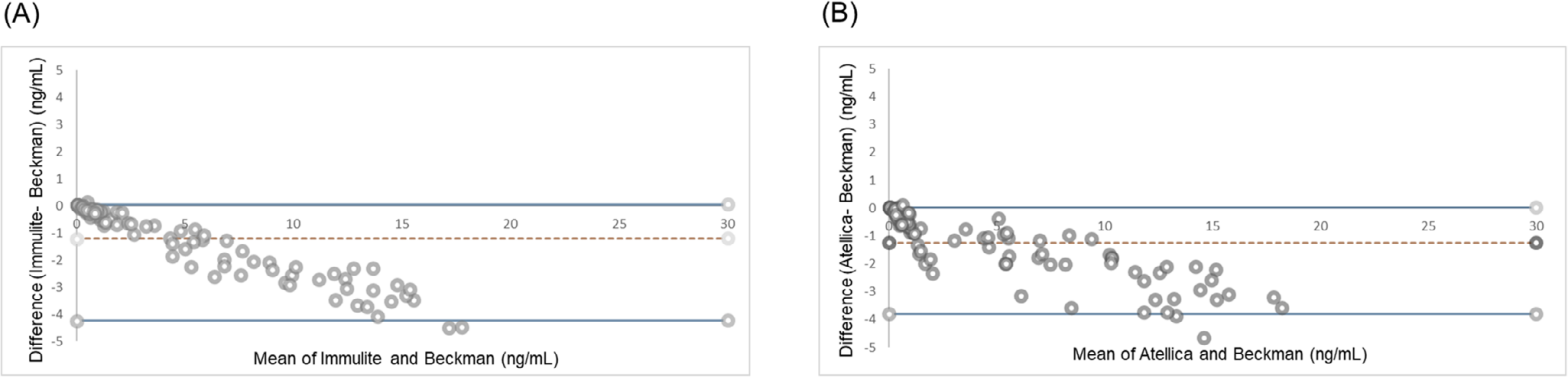
Bland–Altman plot of PSA levels by Beckman and Immulite (A) and by Beckman and Atellica.

Bland–Altman plots showed a significant difference between between PSA‐I and the reference method, PSA‐B, as well as between PSA‐A and PSA‐B (Figure 4A,B). PSA‐I and PSA‐B displayed a mean bias of −1.21 ng/mL (95% confidence interval [CI]: −4.25 to 0.032, *p* < 0.0001). The regression equation was Y = −3.65X + 0.25. The 95% confidence intervals (CIs) for the slope and Y‐intercept were −3.85 to −3.45 for the slope, and −0.13 to 0.62 for the Y‐intercept. PSA‐A and PSA‐B displayed a mean bias of −1.23 ng/mL (95% CI: −3.79 to 0.02, *p* ≤ 0.0001), the regression equation was *Y* = −3.98*X* − 0.26 and 95% CIs for slope and *Y*‐intercept were −4.38 to −3.58 and −0.97 to 0.45, respectively.

### 3.6 Comparability of Well‐Established PSA‐I and PSA‐A With PSA‐B

**FIGURE 5:**
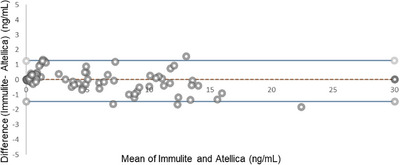
Bland–Altman plot of PSA levels by Immulite and Atellica.

Bland–Altman plots showed no significant difference between the well‐established Siemens PSA‐I and the new PSA‐A (Figure 5). PSA‐I and PSA‐A displayed a mean bias of 0.025 ng/mL (95% CI: −1.44 to 1.28, *p* = 0.93. The regression equation was *Y* = −3.73*X* + 4.15 and with 95% CIs of −3.95 to −3.51 for the slope and 3.28 to 5.02 for the Y‐intercept.

Systematic (constant) and proportional errors were estimated. For PSA‐I versus PSA‐B, the regression equation was *Y* = −3.65*X* + 0.25, with a slope 95% CI of −3.85 to −3.45 and *Y*‐intercept 95% CI of −0.13 to 0.62, indicating the presence of both systematic and proportional bias. For PSA‐A versus PSA‐B, the regression equation was *Y* = −3.98*X* − 0.26, with a slope CI of −4.38 to −3.58 and a *Y*‐intercept CI of −0.97 to 0.45. For PSA‐I versus PSA‐A, the equation was *Y* = −3.73*X* + 4.15, with a slope CI of 3.28–5.019 and an intercept CI of 0.75–1.85.

The mean bias and 95% CIs, derived from Bland–Altman analysis, further quantified the systematic differences between methods: PSA‐I versus PSA‐B: mean bias = −1.21 ng/mL (95% CI: −4.25 to 0.032); PSA‐A versus PSA‐B: mean bias = −1.23 ng/mL (95% CI: −3.79 to 0.02); PSA‐I versus PSA‐A: mean bias = 0.025 ng/mL (95% CI: −1.44 to 1.28).

## 4 Discussion

In this study we found a strong correlation among different PSA assays, with significant variability in tPSA values among three WHO‐calibrated assays from Beckman (PSA‐B) and two assays from Siemens, Immulite (PSA‐I) and the recently developed Atellica (PSA‐A).

The analysis revealed that, when stratifying values by clinically relevant PSA ranges, PSA‐I displayed a good correlation with PSA‐B for values below 2 ng/mL, as well as for values between 2 and 10 ng/mL and above 10 ng/mL. A significant positive correlation was also observed between PSA‐A and PSA‐B in the overall population and across the stratified PSA ranges (<2 ng/mL, 2–10 ng/mL, and >10 ng/mL). The correlation between PSA‐B and PSA‐I was significantly higher than the correlation between PSA‐B and PSA‐A for samples with PSA below 2 ng/mL.

The degree of variability was not wide, as shown by Bland–Altman analysis, suggesting that these assays could be chosen interchangeably. However, the study further showed that the assay choice could impact the detection of biochemical recurrence since since PSA‐I, but not PSA‐A, displayed good correlation with PSA‐B for values below 2 ng/mL. Observed biases and proportional errors could influence clinical decisions, particularly around biopsy thresholds and monitoring of biochemical recurrence, especially for PSA values below 2 ng/mL, where PSA‐A showed reduced concordance with the reference method.

Despite the development of several new biomarkers [14], PSA is currently used to recommend biopsy [15] and to identify recurrence [16], thus, the clinical impact of such interassay variability is relevant as inconsistent results can lead to unnecessary or omitted biopsies and to missed recurrences.

This issue becomes a serious concern when a patient undergoes serial PSA tests using different assays. Some authors showed that clinical laboratories used several different assays [17, 18]. Such diversity may create a scenario where patients could be recommended to undergo a biopsy since they had an initial PSA test at one laboratory and a subsequent one at another. Clinicians do not have detailed knowledge of the technical characteristics of the assay used to measure the patient's PSA level. Patients with PCa must be monitored using the same assay. Thus, it is absolutely necessary that clinicians have a clear idea that PSA values can be dependent on methods for clinical decision‐making [19]. Furthermore, the discrepancies among methods suggested that it could be preferable to use assay‐specific thresholds indicated by manufacturers and/or suggested by clinical guidelines [20].

Our study has several limitations: a small sample size and a study population homogeneous in terms of race and ethnicity. This study was conducted using 104 serum samples collected over a short time period at a single clinical center. Other authors have shown that results could be affected not only by calibration differences but also by ethnicity [21, 22]. While the results provide valuable insights into the comparability of PSA immunoassays, the findings may not be fully generalisable. To strengthen the clinical relevance and statistical robustness, future studies should include a larger and more diverse patient cohort across multiple centres.

Another limitation of this study is the inability to stratify patients by clinical indication. As clarified in the Methods section, serum samples were selected from routine diagnostic testing and included a heterogeneous population of men undergoing PSA testing for various purposes, including initial diagnosis, monitoring, and follow‐up. However, due to retrospective anonymisation of samples, it was not possible to further stratify patients by clinical context. This may have limited our ability to interpret assay performance in specific clinical contexts.

Nevertheless, this study provided preliminary data that may be clinically relevant for patients and clinicians involved in PSA use.

Our findings have potentially relevant clinical implications, particularly in the low PSA range (<2 ng/mL), where assay variability can significantly impact decisions regarding the detection of biochemical recurrence. In this critical range, the Immulite assay (PSA‐I) showed stronger concordance with the reference method (PSA‐B) compared to the Atellica assay (PSA‐A). This suggests that PSA‐I may be more appropriate in clinical settings where higher sensitivity and consistency with the standard reference are essential. Such assay selection could help reduce the risk of false negatives or unnecessary interventions in patients being monitored for early recurrence.

## 5 Conclusions

Our findings suggested that the newly developed Atellica PSA assay showed a strong correlation with the Beckman reference method. However, this assay showed significant inconsistency for PSA values below 2 ng/mL. This discrepancy can impact the early detection of PCa, leading to unnecessary biopsies and missed biochemical recurrences diagnoses. This evidence underscores the need for awareness among physicians regarding interassay variability, recognising that for clinical decision‐making, it may be preferable to (1) use assay‐specific thresholds rather than universal PSA cut‐offs and (2) consistently apply the same assay for each patient.

## Conflict of Interest

The authors declare no conflicts of interest.

## Data Availability

The data that support the findings of this study are available from the corresponding author upon reasonable request.
